# 3D Printed Composite Scaffolds of GelMA and Hydroxyapatite Nanopowders Doped with Mg/Zn Ions to Evaluate the Expression of Genes and Proteins of Osteogenic Markers

**DOI:** 10.3390/nano12193420

**Published:** 2022-09-29

**Authors:** Rebeca Leu Alexa, Andreia Cucuruz, Cristina-Daniela Ghițulică, Georgeta Voicu, Liliana-Roxana Stamat (Balahura), Sorina Dinescu, George Mihail Vlasceanu, Horia Iovu, Andrada Serafim, Raluca Ianchis, Lucian-Toma Ciocan, Marieta Costache

**Affiliations:** 1 Advanced Polymer Materials Group, Department of Bioresources and Polymer Science, University POLITEHNICA of Bucharest, 1-7 Gheorghe Polizu street, 011061 Bucharest, Romania; 2 Department of Biomaterials and Medical Devices, Faculty of Medical Engineering, University POLITEHNICA of Bucharest, 1-7 Gheorghe Polizu street, 011061 Bucharest, Romania; 3 Department of Science and Engineering of Oxide Materials and Nanomaterials, Faculty of Applied Chemistry and Materials Science, University POLITEHNICA of Bucharest, 1-7 Gheorghe Polizu street, 011061 Bucharest, Romania; 4 Department of Biochemistry and Molecular Biology, Faculty of Biology, University of Bucharest, Splaiul Independenței, 050095 Bucharest, Romania; 5 Research Institute of the University of Bucharest, University of Bucharest, 90 Panduri Street, 050663 Bucharest, Romania; 6 Academy of Romanian Scientists, Splaiul Independentei no.54, 050094 Bucharest, Romania; 7 National R-D Institute for Chemistry and Petrochemistry ICECHIM—Bucharest, Splaiul Independentei 202, 6th District, 060021 Bucharest, Romania; 8 Department of Prosthetics Technology and Dental Materials, “Carol Davila” University of Medicine and Pharmacy, 8 Eroii Sanitari Street, 050474 Bucharest, Romania

**Keywords:** hydroxyapatite, 3D printing, GelMA, osteogenesis

## Abstract

As bone diseases and defects are constantly increasing, the improvement of bone regeneration techniques is constantly evolving. The main purpose of this scientific study was to obtain and investigate biomaterials that can be used in tissue engineering. In this respect, nanocomposite inks of GelMA modified with hydroxyapatite (HA) substituted with Mg and Zn were developed. Using a 3D bioprinting technique, scaffolds with varying shapes and dimensions were obtained. The following analyses were used in order to study the nanocomposite materials and scaffolds obtained by the 3D printing technique: Fourier transform infrared spectrometry and X-ray diffraction (XRD), scanning electron microscopy (SEM), and micro-computed tomography (Micro-CT). The swelling and dissolvability of each scaffold were also studied. Biological studies, osteopontin (OPN), and osterix (OSX) gene expression evaluations were confirmed at the protein levels, using immunofluorescence coupled with confocal microscopy. These findings suggest the positive effect of magnesium and zinc on the osteogenic differentiation process. OSX fluorescent staining also confirmed the capacity of GelMA-HM5 and GelMA-HZ5 to support osteogenesis, especially of the magnesium enriched scaffold.

## 1. Introduction

Bone is a complex organ, consisting of collagen and calcium phosphate apatite crystals. It plays crucial physiological roles within the living body (e.g., structural support, physical protection, hematopoietic and immunological functions, mineral storage, and calcium homeostasis) [1,2]. Considering that the significant loss and damage of bone tissue may occur due to trauma, injury, different diseases, or advancing years [1,2,3], the regeneration of lost bone and restoration of its function represents a subject of substantial interest within the research community [3,4]. In an effort to simulate natural bone tissue, numerous bone regeneration strategies have been reported in the literature. These include a combination of various materials (e.g., inorganic materials, natural or synthetic polymers, and composite materials) [5,6] and many designs constructed through different methods (e.g., bioprinting, electrospinning, and emulsion freeze-drying) [7,8,9]. Among these biomaterials, biodegradable hydrogels are receiving increased interest in the biomedical field, particularly in the bone tissue engineering field, due to their excellent biocompatibility, biodegradability, water content, biomimetic, and soft mechanical properties [2,4,10]. As scaffolds with optimal features for bone tissue engineering, aside from the above-mentioned requirements, the biomaterial should ensure a water environment for cells, adequate oxygen, and diffusion of nutrients [11,12]. As hydrogels are cross-linked networks consisting of highly hydrophilic polymers, they can absorb and maintain a high water content. Furthermore, they are highly porous which allows oxygen and nutrients to spread within the scaffolds [11,13,14]. In this context, hydrogels based on natural polymers (e.g., gelatin or its derivative, methacrylate gelatin) that can provide physical support for cells and are capable of promoting new cell development (owing to their intrinsic characteristics of biocompatibility, biomimicry, and RGS) are widely used in bone tissue engineering [4,5,15].

In order to improve the biological properties of hydrogel, particularly the osteoconductivity which is an essential characteristic for biomaterial with possible applications in the processes of bone regeneration, bioactive compounds such as calcium phosphates are usually included in the hydrogel matrix [16,17]. One of the most widely used calcium phosphates with applicability in tissue engineering is HA (Ca_10_(PO_4_)_6_(OH)_2_). This calcium phosphate has gained a leading place in recent years as a biomaterial used in bone implants or fillers. This is due to its properties of biocompatibility, bioactivity, and osteoconductivity, but also due to the crystalline structure similar to the inorganic part of hard tissue, such as bones and teeth [18]. HA ceramics form a strong bond with hard tissue when implanted in the human or animal body [19,20].

Literature studies suggest that the presence of inorganic ions in the structure of hard tissue facilitates regeneration processes, and Ca^2+^ ions in the structure of HA can be substituted with bivalent metal ions, such as Mg^2+^ or Zn^2+^. The presence of these ions can improve the physico-chemical, morphological, and mechanical properties of HA, but also the biological performance for its use in tissue engineering applications [21,22].

The amount of zinc ions in the bone structure depends on the type of bone tissue, age, and sex, but also the nutrition of a person. Literature studies suggest that the ratio of Ca/Zn ions varies depending on the type of bone (trabecular or compact). In the structure of HA, zinc ions prefer the replacement of Ca(II) than Ca(I) ions, being in a more energetically favorable position, and literature studies suggest that the lower ionic radius of zinc ions (0.74 Å) compared to calcium ions (1.00 Å) can lead to a decrease in crystallite size [23,24]. Some of the most important roles of zinc ions are:

- improved antibacterial and anti-inflammatory activity [25].
- in the structure of calcium phosphates, they have shown to induce bone formation and mineralization around the implant, also inducing a decrease in the inflammatory response [26].
- decrease bone resorption due to inhibition of osteoclast [27].
- are of crucial importance in several biological roles, such as the regulation of enzymatic activity [23].

Magnesium is the one of the most common cations in the body; 50–60% exists in hard tissues, less than 1% exists in the blood, and the rest is found in soft tissues. It is essential for regulating many cellular and enzymatic functions. Magnesium ions also play an important role in bone metabolism, and a deficiency of these ions in the structure of hard tissues can lead to a decrease in bone mass [28,29,30]. Recent studies have shown that the presence of magnesium ions plays a key role in the formation of a structure called whitlockite (Ca_9_Mg(HPO_4_)(PO_4_)_6_), which is the second mineral phase in hard tissues. Studies suggest that a higher ratio of whitlockite in the body was detected at an early age, but also in the previous stage of the biomineralization process, which suggests that this mineral has an important influence on the development of hard tissue [31,32]. Some of the most important roles of magnesium ions in the structure of hard tissues are:

- plays an important role in the integrity of the cytoskeleton, through the synthesis of proteins and nucleic acids, by regulating the transport of calcium ions by activating phagocytosis.
- can regulate cell compartments, such as improving cell adhesion and stimulating cell differentiation.
- stimulates bone formation and healing by promoting neovascularization.
- inhibits the function of osteoclasts and promotes the activity of osteoblasts [29].

Several methods of synthesis are used to obtain doped HA. In this study, the authors of this article opted for co-precipitation synthesis, which is one of the most widely used methods of obtaining this doped calcium phosphate due to its ease of use and its ability to obtain a wide range of particle sizes and morphologies [33].

In recent years, focus has shifted to the design of three-dimensional (3D) functional bioconstructs. These can be customized according to a particular application, becoming highly desirable in bone tissue engineering [15,34]. Three-dimensional printing represents a non-invasive, cutting-edge technology that can produce highly scalable and customized artificial structures for personalized medicine, attracting an enormous interest in both academia and clinical practice [34,35,36].

In light of these findings, we constructed 3D composite hydrogels based on methacrylated gelatin (GelMA) reinforced with HA doped with two types of ions (Zn^2+^ and Mg^2+^) as potential scaffolds for bone tissue engineering. The 3D structure of composite scaffolds with three different concentrations of each type of ceramic was achieved through 3D printing, while the mechanical stability of scaffolds was improved through GelMA UV-chemical crosslinking. A detailed structural (FTIR) morphological (SEM), mechanical (nanoindentation), and biological characterization was performed. The results suggest that the 3D printed composite scaffolds can be considered for further investigations as biomaterials with potential applications in bone tissue engineering.

## 2. Materials and Methods

### 2.1. Materials 

#### 2.1.1. Materials for the Obtaining of HA Ceramic Nanopowders Substituted with Magnesium and Zinc

HA ceramic nanopowders substituted with magnesium and zinc ions were synthesized using the precipitation method. The calcium nitrate tetrahydrate (Ca(NO_3_)_2_ · 4H_2_O ≥ 99.0%), dibasic ammonium phosphate ((NH_4_)_2_HPO_4_ ≥ 98%), ammonium hydroxide solution (NH_4_OH, 28.0–30.0% NH_3_), magnesium nitrate hexahydrate (Mg(NO_3_)_2_ · 6H_2_O—99.99%), and zinc nitrate hexahydrate (Zn(NO_3_)_2_ · 6H_2_O ≥ 98%) were purchased from Sigma Aldrich.

The necessary quantities of raw materials for the synthesis of the ceramic nanopowders were determined by performing the calculation to obtain Ca_10−x_M_x_(PO_4_)_6_(OH)_2_, where M is magnesium or zinc ions and x = 2, 5, 10% (sample cod HM2, HM5, and HM10) for magnesium ion concentration and x = 0.1, 0.5, 1% (sample cod HZ1, HZ5, and HZ10) for zinc ion concentration. In the calculation of raw materials, the molar ratio (Ca + Mg or Ca + Zn)/P of 1.67 was respected. The basic principle of the present method for obtaining doped HA nanopowders was also used in a previous paper published by the authors of this article [37].

#### 2.1.2. Materials for Methacrylate Gelatin Synthesis

Gelatine (type B) from bovine skin was purchased from Sigma-Aldrich, St Louis, MO, USA. Methacrylic anhydride (MA) was purchased from Sigma-Aldrich, Goettingen, Germany, and 2-hydroxy-40-(2hydroxyethoxy)-2-methylpropiophenone Irgacure 2959 (I-2959) was purchased from Sigma-Aldrich, Milano, Italy. The raw materials were used as received. Ultrapure water and phosphate-buffered saline (PBS) solution pH = 7.4 were prepared in our laboratory.

#### 2.1.3. Materials for the Determination of Biological Properties

Cell media (Dulbecco Modified Eagle’s Medium low glucose) and supplements (antibiotic antimycotic solution, fetal bovine serum) were purchased from Sigma-Aldrich, Germany. The assays necessary for the biocompatibility assessment (3-(4,5-dimethylthiazolyl-2)-2,5-diphenyltetrazolium bromide (MTT), in vitro toxicology assay kit lactate dehydrogenase (LDH)-based TOX7 assay, Hoechst 33258 solution for fluorescence staining of cell nuclei) and immunofluorescence staining (Triton-X100, paraformaldehyde (PFA) solution) were purchased from Sigma-Aldrich, Germany. The live/dead assay kit and StemPro osteogenesis differentiation kit were purchased from Thermo Fisher Scientific, USA. Goat anti-mouse secondary antibody AlexaFluor 488 (A11029), and goat anti-rabbit secondary antibody AlexaFluor 546 (A11010) were purchased from Invitrogen, USA. Osteopontin (OPN) (sc-21742) and osterix (OSX) antibodies (sc-393325) were purchased from Santa Cruz Biotechnology, Inc. (Dallas, TX, USA) For gene expression experiments, we used TRIzol reagent purchased from Invitrogen, USA, and an iScript cDNA synthesis kit was purchased from BioRad, Hercules, CA, USA.

### 2.2. Methods

#### 2.2.1. Synthesis of Hydroxyapatite Nanopowders

**Figure 1:**
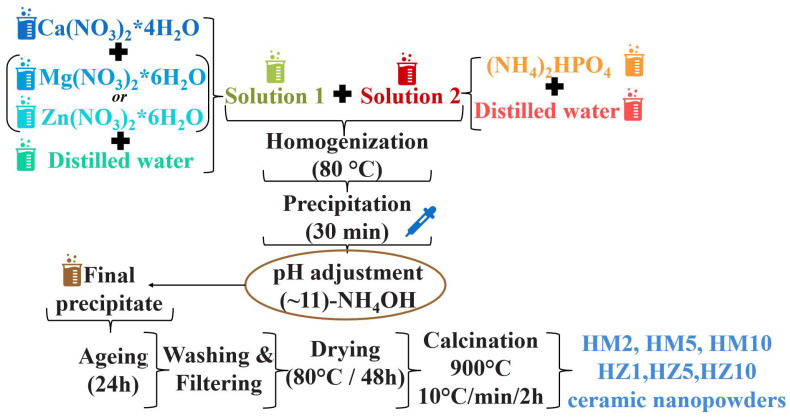
Scheme for HA nanopowders doped with magnesium ions (HM2, HM5, and HM10) and zinc ions (HZ1, HZ5, and HZ10) obtained by precipitation reactions.

A co-precipitation method was used in our laboratory for the synthesis of ceramic HA nanopowders doped with magnesium or zinc ions (Figure 1). The synthesis of HA nanopowders was performed according to the previously reported method by the authors of [37].

The precursors, calcium nitrate tetrahydrate, magnesium nitrate hexahydrate, or zinc nitrate hexahydrate were dissolved in distilled water to obtain solution 1. Dibasic ammonium phosphate was then dissolved in distilled water to obtain solution 2. Solution 2 was homogenized over solution 1 dropwise at 80 °C, resulting in a milky white precipitate.

The pH was then brought to a value of 10–11 in an NH_4_OH solution and was left to mature for 1 day at room temperature. In the last stages of synthesis, in order to obtain the ceramic nanopowders, the precipitate was washed with distilled water, filtered, then dried at 80 °C for 48 h. Finally, the dry powder was calcined at 900 °C.

#### 2.2.2. Synthesis of GelMA

The preparation of Gelatin methacrylate was performed according to the previously reported method by the authors of [35,38].

The first step in obtaining methacrylate gelatin was to dissolve the gelatin in PBS (pH 7.4) at 50 °C. Then, after complete solubilization of gelatin in PBS (~45 min), methacrylic anhydride was added dropwise, which reacted with the functional groups in the gelatin structure for about 2.5 h, under magnetic stirring at 50 °C.

In the final step, dialysis of methacrylate gelatin was performed to remove unreacted methacrylic anhydride. The dialysis process was performed at 40 °C for 5 days using cellulose dialysis bags and distilled water. The obtained GelMA was dried by lyophilization at 0.025 bars for 24 h. 

#### 2.2.3. Synthesis of Composite Materials

Synthesis of the composite materials (GelMA-HMg2, GelMA-HMg5, GelMA-HMg10, GelMA-HZn1, GelMA-HZn5, and GelMA-HZn10) was performed as follows:

Firstly, HA substituted with magnesium/zinc ions in a concentration of 3% *w*/*v* from the total amount of GelMA was dispersed in PBS for 1 h and sonicated for 5 min. Following this, GelMA was added (20% *w*/*v*) and allowed to react for 24 h at 40 °C. After a homogeneous mixture was obtained, Irgacure was added at a concentration of 1% *w*/*v* from the total amount of GelMA and allowed to dissolve under gentle stirring at 40 °C. Finally, the bio-ink was inserted into the 3D printing cartridge [37].

All samples were biologically characterized in order to choose the most compatible Zn/Mg concentration for cell viability.

After a fixed concentration of HA doped with magnesium/zinc ions was selected (sample HM5, HZ5), the optimization of the GelMA concentration was followed in order to obtain the suitable 3D printing bio-ink. 

To obtain the appropriate printing paste, 3 concentrations of GelMA were used (20%, 25%, and 30% *w*/*v*) and the same working method described above was followed, using a concentration of 3% *w*/*v* of HM5 and HZ5.

### 2.3. Characterization Techniques 

#### 2.3.1. X-ray Diffraction (XRD)

X-ray diffraction (XRD) was used to determine the composition and crystallinity degree of dry precipitates and ceramic nanopowders. This was performed using a Shimadzu XRD 6000 diffractometer (Shimadzu, Kyoto, Japan), with Ni-filtered CuK α radiation (α = 1.5406 Å), 2θ in 20–70° range, with a scan step of 0.02° and a counting time of 0.6 s/step. The average diameter of crystallite, D (nm) was calculated using the Scherrer equation (Equation (1)):

(1) $$D=\frac{K\lambda }{\beta \cos\theta }$$

where D is the average particle diameter (Å), K is a constant (0.94), λ is the X-ray radiation wavelength (1.5406 Å), θ is the peak angle, and β is the width at half maximum (FHWM) of the respective XRD peak [37,39].

#### 2.3.2. Brunauer–Emmett–Teller (BET)

The Brunauer–Emmett–Teller (BET) analysis technique was used to determine the morphological composition—the specific surface area and the pore size dimensions—which was performed on a Micrometrics Gemini V2 model 2380 (Micromeritics Instruments Corporation, Norcross, GA, USA). The adsorption isotherms were obtained by measuring the amount of gas adsorbed across a wide range of relative pressures at a constant temperature (N2, 77 K and pressure between 780 and 7.8 mmHg). Conversely, desorption isotherms were achieved by measuring the gas removed as pressure is reduced [37].

#### 2.3.3. Laser Diffraction Granulometer

A Malvern Mastersizer 2000 laser diffraction granulometer (Malvern Instruments, Malvern, UK) was used to determine the particle size distribution of HA nanopowders (HM5 and HZ5) [37].

#### 2.3.4. Scanning Electron Microscopy SEM

Scanning electron microscopy (SEM) using a Quanta Inspect F50 FEG scanning electron microscope was used to determine the microstructure of composite materials, with a resolution of 1.2 nm (Thermo Fisher, Eindhoven, The Netherlands). The composite materials were covered with a thin gold layer [37,40].

#### 2.3.5. FTIR Spectrometry

FTIR spectra were recorded on a Bruker VERTEX 70 instrument, using the total reflection attenuation module (ATR) at a resolution of 4 cm^−1^ and range of 600–4000 cm^−1^, and the final spectra represented the average of 32 scans [37].

#### 2.3.6. Printability

3. A Discovery ™ V6.1 bioprinter (RegenHU, Villaz-St-Pierre, Switzerland) was used to obtain the 3D printed scaffolds. 

In order to design the scaffold architecture, BIOCAD software was used. The direct dispensing print head of the 3D bioprinter was selected to print the bio-inks. 

For establishing the printing parameters, cylindrical nozzles of 23G and 25G were interchanged, and printing speeds in the range of 4–10 mm/s and pressures in the range of 70–250 kPa were varied. Finally, bio-inks were printed at room temperature and scaffolds obtained were UV cured at 360 nm [37].

#### 2.3.7. Swelling Degree and Degradability of the 3D Printed Hydrogel Based on GelMA

To determine the water absorption capacity of the 3D printed scaffolds, swelling analyses were performed in triplicate. Furthermore, all samples were analyzed from the degradation point of view. These studies were performed because the water absorption or degradation capacity of scaffolds affect cell proliferation as well as tissue regeneration.

To perform the swelling analyses, 3D printed scaffolds were lyophilized, weighed, and immersed in PBS. After predetermined immersion times, scaffolds were weighed [37]. 

The water retention was calculated using Equation (2).

(2) $$\mathrm{Swelling}\;\mathrm{degree}\;(\%)=\frac{\mathrm{Ww}-\mathrm{Wd}\;}{\mathrm{Wd}}\;\;\times \;100$$

where Ww = wet weight and Wd = dry weight.

To be characterized from the degradation point of view, 3D printed samples were immersed in PBS for 24 h, then lyophilized and weighed. 

The degradation capacity of scaffolds was calculated using Equation (3).

(3) $$\mathrm{Degradation}\;\mathrm{degree}\;(\%)=\frac{W0-\mathrm{Wd}}{\mathrm{Wd}}\times 100$$

where Wo = initial weight and Wd = dry weight. 

#### 2.3.8. Micro-Computer Tomography (µ-CT)

The micro-computer tomography (µ-CT) investigation was performed using the high-resolution Bruker CT 1272 equipment. With a 50 kV source voltage, a 130 A current intensity, and a 450 ms exposure period in each frame, the scanning was undertaken without a filter [37]. The samples were rotated 180° with a 0.2° rotation step throughout the scanning process. For each unique slice, the image was constructed by averaging 3 frame acquisitions. For each of the 3 examples, the image pixel size was set at 6 µm.

Tomograms were recovered from raw data using the Bruker NRecon 1.7.1.6 program (Kontich, Belgium). Smoothing was set to 1, with beam hardening correction set to 7–9, ring artefact reduction set to 5, and beam hardening correction set to 7–9. To visualize reconstructed tomograms, CTVox (Bruker) was employed, and CTAn 1.17.7.2 was used for sample analysis (Bruker, Kontich, Belgium). For each material, a single volume-of-interest (VOI) dataset was extracted, containing 100% of the scanned area.

In CTAn, the VOIs were subjected to an image-processing task list that included thresholding (to separate the specimen walls from its pores), de-speckling (to remove residual scanning artefacts), and 3D analysis for the numerical quantification of total porosity (tp), structure separation (pore dimensions), and structure thickness (wall thickness) were studied. The tomogram pixels were binarized after thresholding (all the pixels associated to the solid sample were converted in white while the rest were in black, depicting the pores within the sample). Quantitative analysis was performed using a 6 µm scanning resolution to identify the width domains of the solid prints/specific porosity by computing the object feature size comparable to respective white/black 3D pixels (voxels) [37]. The accumulated readings are supplied at predetermined intervals, starting with the scanning resolution and increasing by thrice (e.g., [6–18 m], [18–30 m], [30–42 m], [42–54 m). The corresponding values of these domains were partially grouped for convenience in this report.

CTAn also allows for the separation of size-specific object characteristics, such as unique pore domains. To do this, the pictures were inverted after binarizing the dataset; this technique allowed for the display and measurement of the pores as solid objects, as well as their separation depending on specified boundaries. This procedure was used to extract the 3D tomograms of the pores in the following intervals: [6–100 µm], [100–200 µm], [200–300 µm], etc. for 20%GelMAHZn5, 25%GelMA-HZn5 and 30%GelMA-HZn5 and 20%GelMAHMg5, 25%GelMAHMg5, and 30%GelMAHMg5. To further understand the interface and dispersion of the pore network, CTVox was utilized to display the pore tomograms loaded into the 3D solid print [37].

#### 2.3.9. Mechanical Properties of 3D-Printed Scaffolds

The mechanical properties of the 3D-printed scaffolds based on GelMA-nanofiller were studied using a nanoindentation technique and a rheological analysis. 

Rheological analyses were performed using a Kinexus Pro Rheometer (Malvern, Worcestershire, UK) equipped with a Peltier element for precise temperature control and 20 mm parallel-plate geometry. The frequency sweep measurements were performed at a stress of 5 Pa for all samples within the frequency range of 0.01–10 Hz. 

Using the rheological results, the crosslinking density was calculated using Equation (4) [41].

(4) $$\mathrm{Crosslinking}\;\mathrm{density}\;\nu =\frac{G\prime }{\mathrm{RT}}$$

- G’—the storage modulus
- R—represents the gas constant
- T—the temperature at which G’ was measured (298.15 K).

#### 2.3.10. Nanoindentation

Nanoindentation analyses were performed using a Nano Indenter^®^ G200 (Santa Clara, CA, USA). Tests were performed in triplicate using a G-Series DCM CSM Flat Punch Complex Modulus Gel. Using this method, the storage modulus G’ and loss modulus G’’ were determined at a predefined frequency of 10 Hz [37]. 

#### 2.3.11. In Vitro Biocompatibility Evaluation of 3D Scaffolds

To evaluate the influence of the magnesium or zinc on bone tissue engineering, the GelMA-HAp-based scaffolds were evaluated for their biocompatibility in contact with murine preosteoblasts from the MC3T3-E1 cell line. The cells were cultivated in Dulbecco’s Modified Eagle Medium (DMEM), supplemented with 1% antibiotic-antimycotic and 10% fetal bovine serum (FBS), and stored at 37 °C, 5% CO2. The scaffolds were sterilized by exposure to UV light, swollen using culture media and cut into pieces with a diameter of 1 cm^2^. Cells from the MC3T3-E1 cell line were trypsinized, counted, and seeded on materials at 2 × 10^5^ cells/cm^2^ density through cell suspension, distributed over the composites, and incubated in standard conditions until the assays were performed.

A biocompatibility assessment was accomplished at 2 and 7 days post-seeding, employing both quantitative (MTT and LDH) and qualitative assays (live/dead fluorescent staining). To assess if murine preosteoblasts maintain their metabolic activity in contact with GelMA-HAp enriched scaffolds, a methylthiazolyldiphenyl tetrazolium bromide (MTT) assay was employed. After discharging the culture media, the constructs were incubated with 1 mg/mL MTT working solution for 4 h at 37 °C. The obtained violet formazan crystals were then solubilized with isopropanol and the absorbance of the final product was measured at 550 nm using a FlexStation 3 Spectrophotometer (Molecular Devices, San Jose, CA, USA).

In order to assess if the scaffolds exhibit significant toxicity on MC3E3-E1, an in vitro toxicology assay kit lactate dehydrogenase-based assay was performed, following the manufacturer’s instructions. The obtained solution was measured at 490 nm using a FlexStation 3 Spectrophotometer. The quantity of LDH released in culture media was directly proportional with the percentage of dead cells.

Live/dead fluorescent staining was accomplished using a live/dead kit in order to establish the ratio between live cells and dead cells. The assay provided two fluorescent dyes which mark live cell (calcein acetomethoxy) and dead cell nuclei (ethidium bromide homodimer) in green and red fluorescence, respectively. The solution was prepared following the manufacturer’s instructions and the 3D scaffolds were incubated for 1 h in dark conditions. The images were obtained using a confocal microscope Zeiss 710 and processed using Zeiss Zen software. Quantification of the fluorescence for both live and dead cells was obtained using ImageJ software.

#### 2.3.12. Osteogenic Gene and Protein Expression Evaluation 

The capacity of MC3E3-E1 to undergo osteogenesis when cultivated on GelMA-HAp scaffolds was evaluated. After 24 h of cell culture, a commercially available cocktail of osteogenic inducers was added to the 3D systems. The media was renewed every 3 days and the osteogenic differentiation process was monitored for 28 days. 

OPN and OSN (osteogenic markers) gene expression was investigated using real-time PCR. The RNA was isolated from the 3D printing scaffolds using TRIzol Reagent. Total RNA was investigated for purity and concentration using a NanoDrop spectrophotometer (ThermoScientific, Waltham, MA, USA) and the RNA integrity number (RIN) was determined using an Agilent 2100 BioAnalyzer (Agilent Technologies, Santa Clara, CA, USA). Complementary DNA (cDNA) was synthesized using an iScript cDNA Synthesis kit (BioRad, USA) and amplified by PCR using a Veriti 96-Well Thermal Cycler from Applied Biosystems. The qPCR was performed using the SYBR Green method and ViiA7 equipment (ThermoScientific, USA). The expression of GAPDH was used as reference gene.

Protein expression of OPN and OSX was analyzed by immunofluorescent staining coupled with confocal microscopy. The samples were fixed with a 4% PFA solution for 1 h and permeabilized with 0.1% Triton X100 solution in 2% BSA for 20 min at 4 °C. The samples were then incubated overnight with mouse monoclonal OPN and rabbit polyclonal OSX, and afterwards with goat anti-mouse secondary antibody AlexaFluor 488 and goat anti-rabbit secondary antibody AlexaFluor 546, respectively, for 1 h at 4 °C. Cell nuclei were stained with Hoechst 33258 solution and the samples were visualized using a confocal microscope Zeiss 710 and processed using Zeiss Zen software.

#### 2.3.13. Statistical Analysis 

The obtained results were statistically analyzed using GraphPad Prism software, one-way ANOVA method and the Bonferroni algorithm. All data were indicated as mean ± SD of *n* = 3 experiments and values were considered significant where *p* < 0.05.

## 3. Results and Discussion

### 3.1. Characterization of Ceramic Powders

#### 3.1.1. X-ray Diffraction (XRD)

**Figure 2:**
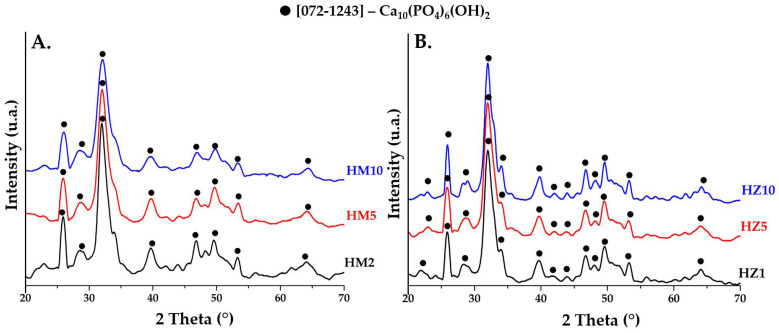
XRD patterns of precipitates obtained after the drying process at 80 °C for (**A**) powders doped with magnesium (HM2, HM5, and HM10) and (**B**) powders doped with zinc (HZ1, HZ5, and HZ10).

Figure 2A shows the mineralogical composition of the precipitates obtained after the drying process doped with magnesium ions (HM2, HM5, HM10) and Figure 2B doped with zinc ions (HZ1, HZ5, HZ10). Both nanopowders doped with magnesium and zinc have a crystalline character, typical for HAp with a hexagonal structure, having the space group P63/m. The main peaks of the mineral phase are positioned at 2θ at 25.9° (0 0 2), 32.2° (2 1 1), 33.9° (2 0 2), 39.7° (1 3 0), 46.7° (2 2 0), and 49.5° (2 1 3) according to JCPDS standard no. 072-1243. The spectra from the dry nanopowders are similar for all compositions. No significant changes are noticed in the case of the dry precipitates, except perhaps a slight decrease in the intensity of the diffraction peaks with increasing dopant concentration in samples doped with magnesium ions. Instead, a slight increase in crystallinity can be observed in the case of samples doped with zinc ions, compared to the powder doped with magnesium ions.

**Figure 3:**
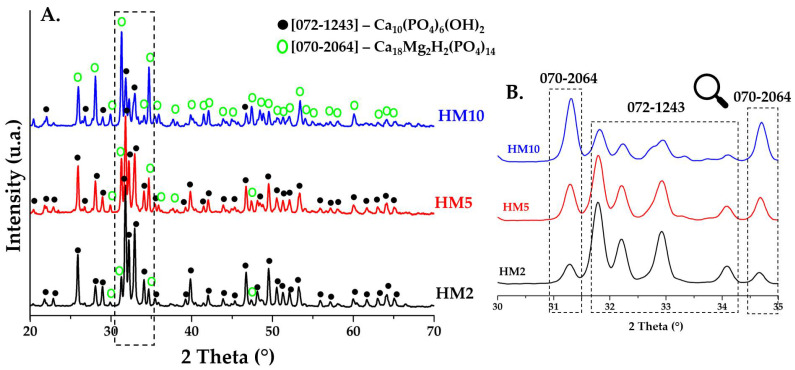
XRD patterns of ceramic powders obtained after calcination heat treatment at 900 °C for HM2, HM5, and HM10.

The diffraction patterns of HA powders doped with magnesium ions and heat treated at 900 °C are shown in (Figure 3A 2θ in the range 20–70, Figure 3B 2θ in the range 30–35). The major phase in doped powders (HM2 and HM5) is HA with a hexagonal crystalline structure (P63/m), according to the characteristics of the main diffraction peaks positioned at approximately 2θ = 25.9° (0 0 2), 31.7° (2 1 1), 32.2° (1 1 2), and 32.8° (3 0 0), in accordance with JCPDS standard no. 072-1243. For the powder doped with a higher percentage of magnesium ions (HM10), the majority phase found is that of whitlockite (Ca_18_Mg_2_H_2_(PO_4_)_14_), with a rhombohedral crystalline structure (R3c), according to the characteristics of the main diffraction peaks positioned at approximately 2θ = 25.9° (1 0 10), 27.9° (2 1 4), 31.3° (0 2 10), and 34.6° (2 2 0) according to JCPDS no. 070-2064. This aspect is obvious in the diffraction pattern in Figure 3B, where the main diffraction peak of the whitlockite identified at 2θ = 31.3° increases in intensity as the dopant concentration in the HA ceramic powder increases, becoming the major phase in the HM10 powder. The crystalline structure of whitlockite is also identified in ceramic powders with a lower percentage of magnesium (HM2 and HM5), but as a minor phase. Studies [19,31,32] suggest that the presence of magnesium ions in the structure of HA leads to the formation mineralogical phase of whitlockite as a majority or secondary phase. This brings an advantage to the final biomaterial, as it has been reported that a mixture of the two phases can lead to an increased capacity for osteogenesis, aspects that are also confirmed in the present study. The ease of HA doping also comes from the fact that Mg^2+^ ions have an ionic radius of 0.72 Å, less than that of calcium ions at 1.00 Å, which allows them this substitution.

**Figure 4:**
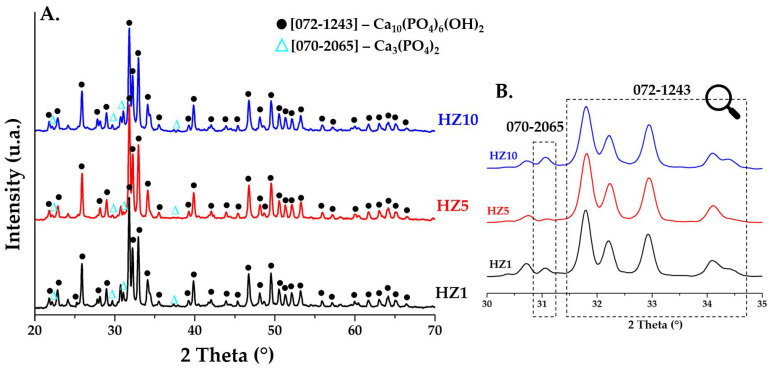
XRD patterns of ceramic powders obtained after calcination heat treatment at 900 °C for HZ1, HZ5, and HZ10.

Figure 4A (2θ in the range 20–70) and Figure 4B (2θ in the range 30–35) shows the diffraction patterns for ceramic HA powders (HZ1, HZ5, and HZ10) doped with zinc ions and sintered at 900 °C. In all three samples of zinc ion-doped HA, a hexagonal crystalline structure (P63m) is identified as the major phase according to the main diffraction peaks of 2θ = 25.9° (0 0 2), 31.7° (2 1 1), 32.2° (1 1 2), and 32.8° (3 0 0) in accordance with JCPDS standard no. 072-1243. The other phase identified is β—tricalcium phosphate (β-TCP) with a rhombohedral crystalline structure (R3c), with the main peaks identified at 2θ = 21.8° (0 2 4), 29.6° (3 0 0), 31° (0 2 10), and 37.3° (1 2 11) in accordance with JCPDS standard no. 070-2065.

As observed in the case of doping with magnesium ions, the substitution of Ca^2+^ ions with Zn^2+^ ions is easily accomplished as the ionic radius of zinc ions is 0.74 Å, and studies suggest [23,26,27] that this substitution leads to a slight decrease in the size of the crystallite. To observe changes in crystallite size, the Debye–Scherrer equation was used and the average crystallite diameter was calculated (Table 1). Thus, the values obtained for all six samples do not show significant differences. In samples doped with magnesium ions, the values increase slightly, and in the case of samples doped with zinc ions, the average size is reduced very slightly, aspects that are noticed in all diffraction patterns in Figure 3 and Figure 4. 

#### 3.1.2. Laser Granulometry

**Figure 5:**
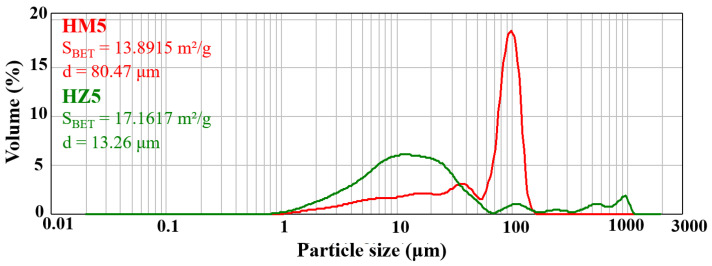
Granulometric distribution of ceramic powder. The red line indicates the magnesium ion doped HA (HM5) and the green line indicates the zinc ion doped HA (HZ5).

To characterize the specific surface area of BET and particle size distribution, ceramic HA nanopowders substituted with magnesium ions (HM5) and zinc ions (HZ5) were investigated using a laser diffraction particle size analyzer (Figure 5). For HM5 powders (Figure 5—red line), a bimodal distribution of powder particle size can be observed, presenting a value of 80.47 μm of the average particle size and 13.89 m^2^/g for the specific BET area, which shows that the powder has a high fineness. In the case of zinc ion-doped HA powder (HZ5) (Figure 5—green line), a multimodal powder particle size is identified with a value of 13.26 μm for the average particle size and 17.16 m^2^/g for the specific BET area, which indicate that this type of powder also has a high fineness. 

The bimodal or multimodal shape of particle size distribution may also suggest a high tendency for the powders to form agglomerates.

### 3.2. Characterization of Composite Materials

#### 3.2.1. Fourier Transform Infrared Spectroscopy (FTIR)

**Figure 6:**
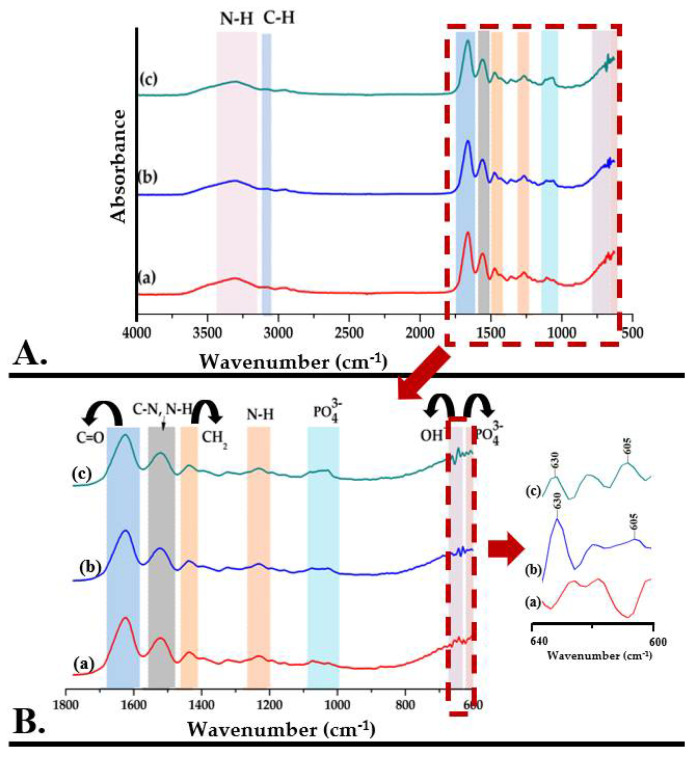
((**A**,**B**)—zoom) FTIR spectra for GelMA (a), GelMA-HMg5 (b), and GelMA-HZn5 (c).

Figure 6 shows the FTIR spectra of methacrylate gelatin, but also of composite materials—GelMA-HMg5 and GelMA-HZn5.

The Fourier transform infrared spectrum of GelMA (Figure 6A(a)) shows the specific absorption bands for the functional groups in the gelatin structure, identifying the peptide bonds (-NH-CO)-) and indicating that in the methacrylation step, the bonds between the amino acids in the structure of the primary proteins were not affected. The main absorption bands and their assignments are presented in Table 2.

The Fourier transform infrared spectrum of composite materials—GelMA-HMg5 and GelMA-HZn5 (Figure 6A(b,c))—shows the overlap of the peaks characteristic for the mineral phase with those of methacrylate gelatin. The wide peak from 3100–3500 cm^−1^ and the one from 630 cm^−1^ are attributed to the stretching vibrations of the OH^-^ groups. The absorption bands specific to HA are also identified by the presence of peaks from 603, 1033 cm^−1^ and 1082 cm^−1^, which are characteristic of the group PO_4_^3−^ (ʋ_4_), PO_4_^3−^ (ʋ_2_), and PO_4_^3−^ (ʋ_3_), respectively [42,43,44].

#### 3.2.2. Scanning Electron Microscopy (SEM)

**Figure 7:**
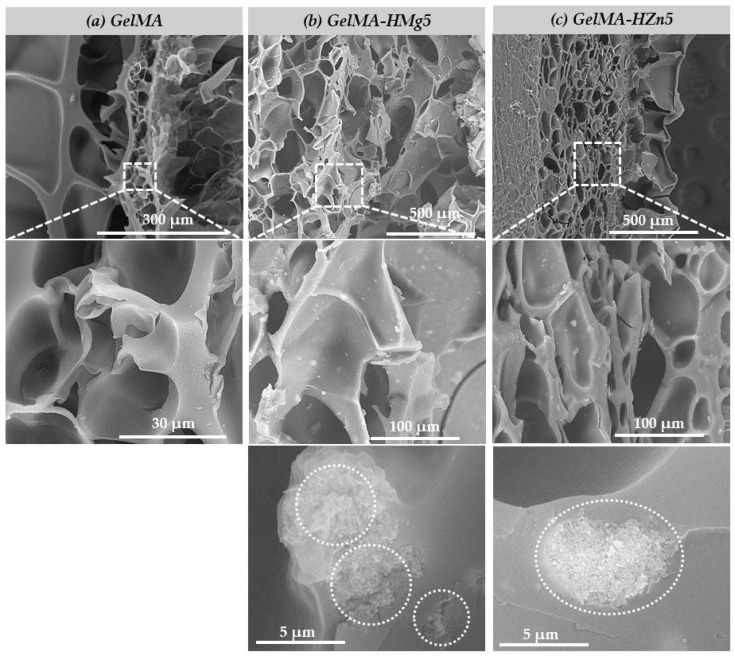
SEM micrographs of samples (**a**) gelatine methacrylate (GelMA), (**b**) GelMA with magnesium (GelMA-HMg5), and (**c**) GelMA with zinc (GelMA-HZn5).

Figure 7 shows the SEM micrographs of the samples of (a) GelMA, (b) GelMA-HMg5, and (c) GelMA-HZn5. Scanning electron microscopy images show for all three types of material a porous microstructure, with evenly distributed, interconnected pores, a microstructure that can be beneficial when used in bone regeneration applications. Also, the microstructure of methacrylate gelatin is ideal for the incorporation of ceramic powders doped with magnesium and zinc ions, and the interconnected pores are beneficial for the migration and adhesion of a high cell density [45,46]. The scanning electron microscopy images in Figure 7b,c shown on GelMA-HMg5 and GelMA-HZn5 composites show the presence of nanoparticles on the surface of the GelMA, and at higher magnifications, these nanoparticles are also present in the mass of GelMA. HA nanopowders (HM5 and HZ5) are uniformly distributed in the methacrylate gelatin matrix, due to the conditions for obtaining the composite materials (described in the method for obtaining in Section 2.2.3), thus resulting in a microstructure of the composite material with uniformly distributed pores, which are interconnected and have different dimensions, as can be seen in the microscopy images. In the case of ceramic powders (HA doped with magnesium ions (HMg5)) the particles present dimensions in the range of 70–140 nm in the form of aggregates, and for HA substituted with zinc ions (HZn5), they have dimensions in the range 50–120 nm, also with aggregate shapes.

**Figure 8:**
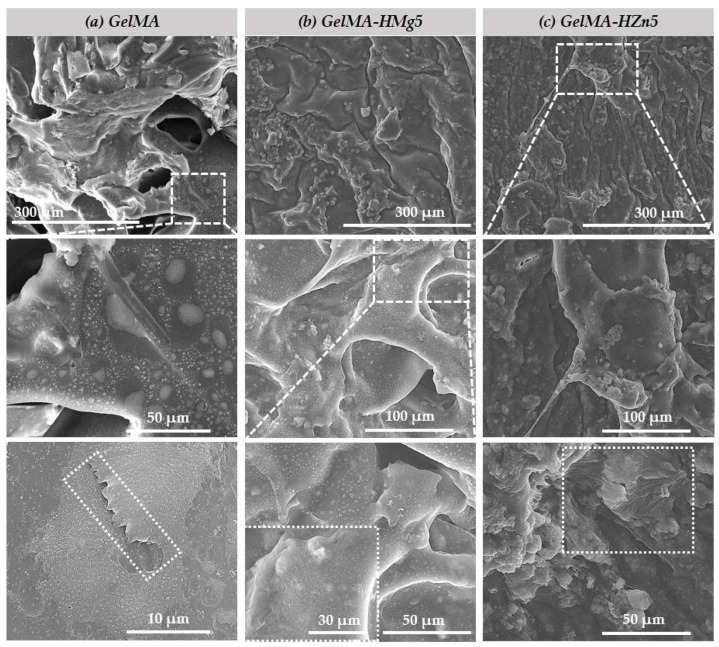
SEM micrographs of (**a**) gelatine methacrylate (GelMA), (**b**) gelatine methacrylate with magnesium (GelMA-HMg5) and (**c**) gelatine methacrylate with zinc (GelMA-HZn5) composites in contact with cell.

The materials obtained, both methacrylate gelatin (GelMA) and composite materials (GelMA-HMg5 and GelMA-HZn5), were seeded with a density of murine preosteoblasts type 2 × 105 cells/cm^2^ from the MC3T3-E1 cell line and subsequently investigated using SEM to observe their microstructure and the interaction of the cells in association with the studied materials (Figure 8). We can see that in all three types of materials, the presence of the attached cells (MC3T3-E1) is uniform on the samples. It can also be observed that some of the cells caught on the surface of the materials, and as other studies show [47], an important factor for good cell adhesion is the roughness of the material. As shown in the studied samples, the cells are forming filopodia, which allows for the anchoring of material and subsequently leads to a better spread in their entire microstructure.

### 3.3. Characterization of the 3D Printed Hydrogel Scaffolds 20%GelMA-3%HAP-Mg, 25%GelMA-3%HAP-Mg, 35%GelMA-3%HAP-Mg, 20%GelMA-3%HAP-Zn, 25%GelMA-3%HAP-Zn, and 35%GelMA-3%HAP-Zn

#### 3.3.1. Printability

The main objective of this research study was to design 3D hydrogel scaffolds based on GelMA with possible application in bone regeneration. In this respect, following the biology results, nanocomposite hydrogels based on gelatine methacrylate were synthesized and 3D printed at room temperature. Three different concentrations of GelMA were used (20%, 25%, and 30%) and two different nanoparticles (HAPMg5 and HAPZn5). Six 3D-printable materials were obtained through their combination.

In order to settle the optimal 3D printing parameters, variable needle dimensions (0.25 mm, 0.33 mm), printing speeds (2–10 mm/s), and pressures (100–250 kPa) were investigated.

As the apparent rheology of synthesized 3D printing inks is induced by the concentration of the polymer and not by the reinforcing agent (HAPZn5, HAPMg5), the printing parameters and the properties of the scaffolds obtained varied only depending on the polymer concentration. For the hydrogel based on 20%GelMA-anorganic filler and 1%Irgacure, a pressure of 105 kPa, 10 mm/s speed, and a needle of 0.25 mm were used.

**Figure 9:**
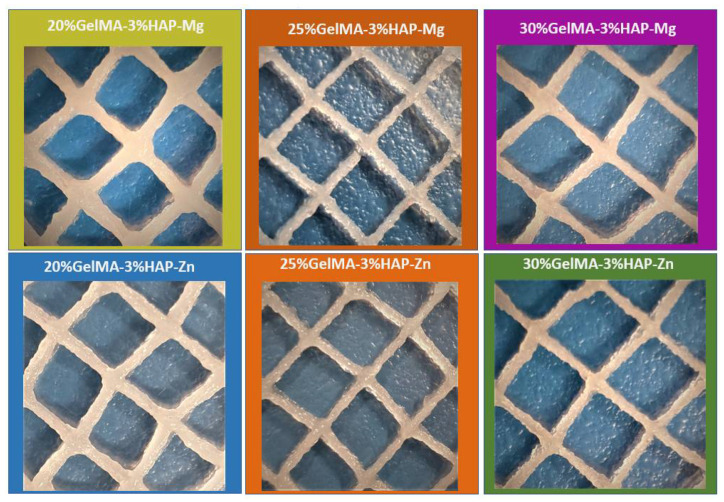
3D printed hydrogel scaffolds: 20%GelMA-3%HAP-Mg, 25%GelMA-3%HAP-Mg, 35%GelMA-3%HAP-Mg, 20%GelMA-3%HAP-Zn, 25%GelMA-3%HAP-Zn, and 35%GelMA-3%HAP-Zn.

Bio-inks presented stability during the printing process, but the final filaments maintained their structural integrity only up to seven layers (Figure 9). As the number of layers increased, the filaments became larger and spread more on the glass slides on which they were printed. 

When the GelMA concentration increased from 25% to 30%, while maintaining the same initiator concentration, the printing parameters had to be modified according to the viscosity of the formulation, and increased pressure and greater needle diameter were used. The bio-ink based on 25%GelMA-anorganic filler was printed using a needle of 0.25 mm, a pressure of 125 kPa, and 10 mm/s speed, and the bio-ink-based 30%GelMA-anorganic filler was printed using a needle of 0.33 mm, a pressure of 130 kPa, and 6 mm/s speed. Both materials kept their architecture for up to 7–10 layers (Figure 9). 

Finally, after the printing process, printed samples were photo-cured using UV light (365 nm with a maximal optical output power of 500) for five minutes [35]).

#### 3.3.2. Swelling Degree and Degradability of the 3D-Printed Bio-Inks Based on GelMA with Different Reinforcer Agents

Hydrogels are the most studied class of biomaterials, with applications in the tissue engineering domain owed to their ability to absorb large quantities of water, as water is a major component of the human body.

To study the hydrophilic/hydrophobic behavior of the bio-inks obtained in this study, swelling degree analyses were made on the UV crosslinked samples obtained by 3D printing. 

**Figure 10:**
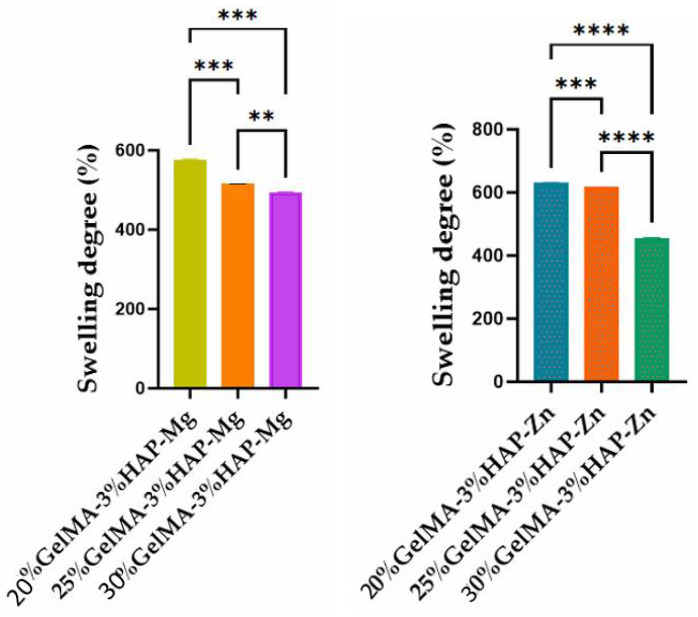
Swelling degree of GelMA-inorganic filler. Statistical significance: **** *p* < 0.0001, *** *p* < 0.001, and ** *p* < 0.01.

Depending on the concentration of GelMA used and the inorganic agent added, the samples showed variations in hydrophilicity; with increasing GelMA concentration, the hydrophilicity of the polymer decreased, along with the degree of swelling (Figure 10). This phenomenon could be due to the presence of a greater amount of hydrophobic methacrylate groups from the GelMA structure [35] when an increased concentration is used. For the samples obtained based on GelMA-Zn, a higher degree of swelling was obtained when compared with GelMA-Mg, most probably due to a higher hydrophilicity induced by Zn.

**Figure 11:**
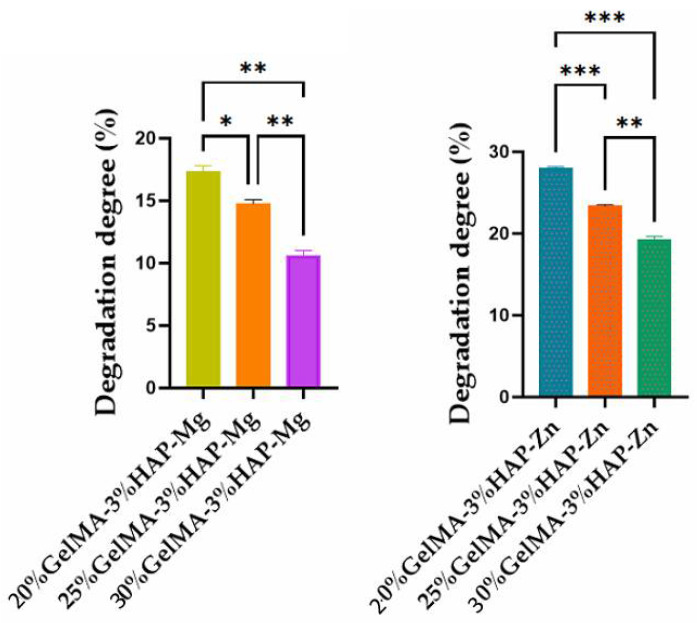
Degradation degree of GelMA-inorganic filler. Statistical significance: *** *p* < 0.001, ** *p* < 0.01, and * *p* < 0.1.

The concentration of GelMA used also influenced the degree of degradation of the samples; therefore, as the gelatin methacyloyl concentration increases, the degree of degradation decreases. This characteristic can be justified by the denser internal structure which generates a reduced porosity in the scaffolds (Figure 11).

#### 3.3.3. Mechanical Properties of the 3D-Printed Scaffolds

Rheological and mechanical analyses were performed on equilibrium swollen samples. 

The mechanical properties of 3D-printed scaffolds based on GelMA-nanofiller was investigated using a nanoindentation technique and rheological analysis. 

**Figure 12:**
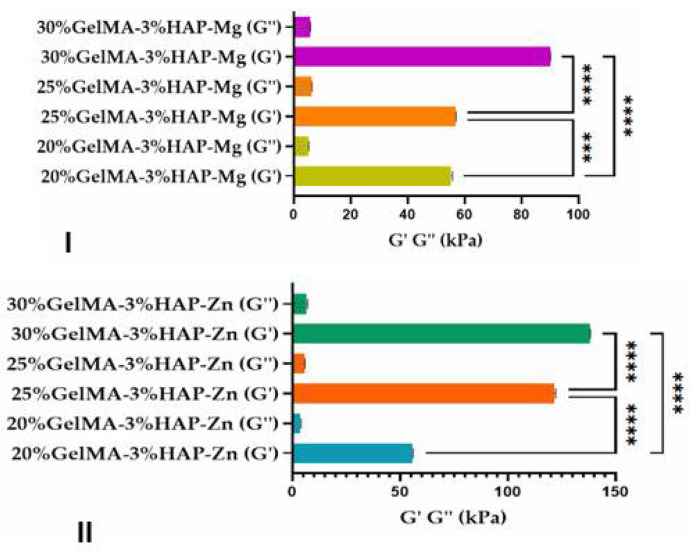
Storage and loss moduli of the GelMA-inorganic filler **((I)—HA doped with magnesium ions and (II)—HA doped with zinc ions)** determined by the nanoindentation technique. Statistical significance: **** *p* < 0.0001.

The nanoindentation analysis, presented in Figure 12, demonstrated that the scaffolds obtained after photopolymerization were crosslinked, and all samples presented a storage modulus higher than the loss modulus (G’ > G’’). Furthermore, all tests revealed that higher concentrations of GelMA improved the mechanical properties of the final scaffolds obtained by 3D printing (Figure 12). Nanoindentation results indicate that at higher GelMA concentrations, almost double the storage modulus values were obtained for the samples reinforced with Zn nanoparticles when compared with the ones obtained with Mg. 

**Figure 13:**
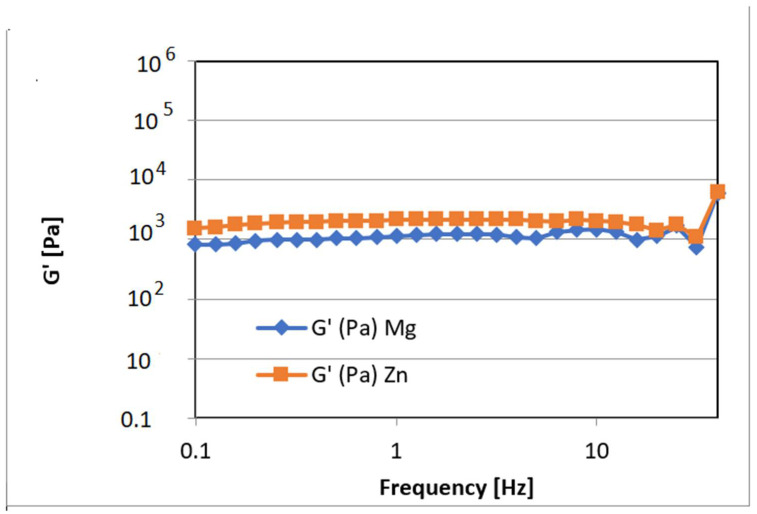
Storage modulus for crosslinked hydrogel based on the GelMA-inorganic filler as functions of frequency measured at 25 °C under a stress of 5 Pa.

Additionally, using rheological tests for samples based on 25%GelMA-Mg and 25%GelMA-Zn, the crosslinking density was calculated. Tests were performed at 25 °C under a stress of 5 Pa, considering the storage modulus of the crosslinked samples as functions of frequency. From the calculated values, it was observed that the crosslinking density was dependent on the nanofiller used: 25%GelMA-Mg exhibited a crosslinking density of 65.9 mol%, whereas 25%GelMA-Zn exhibited a crosslinking density of 116 mol% (Figure 13). 

These results corelated with micro-computer tomography analyses that showed a higher porosity in the samples based on GelMA-Mg than in samples based on GelMA-Zn. This indicates that materials containing Zn exhibit higher mechanical properties than materials containing Mg, most likely due to the more compact structure of the bio-ink containing Zn.

#### 3.3.4. Micro-Computer Tomography (µCT)

**Figure 14:**
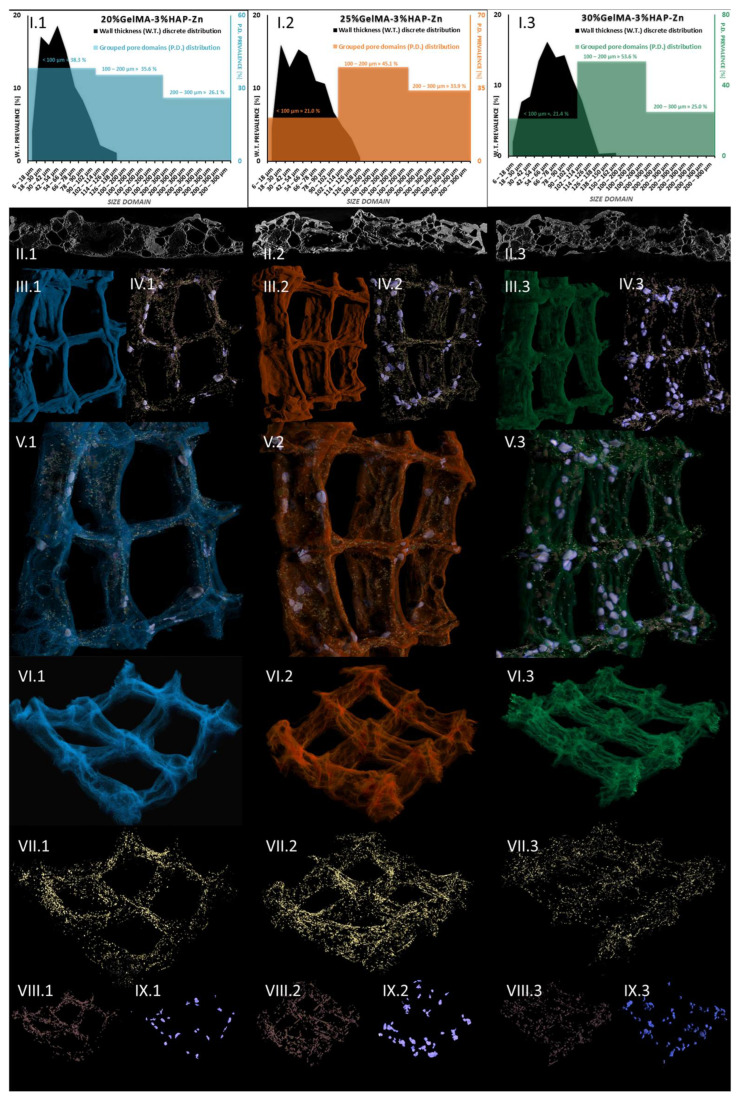
Micro-CT images of: (1 blue) 20%GelMA-3%HAP-Zn, (2 orange) 25%GelMA-3%HAP-Zn, and (3 green) 30%GelMA-3%HAP-Zn 3D-printed composites.

**Figure 15:**
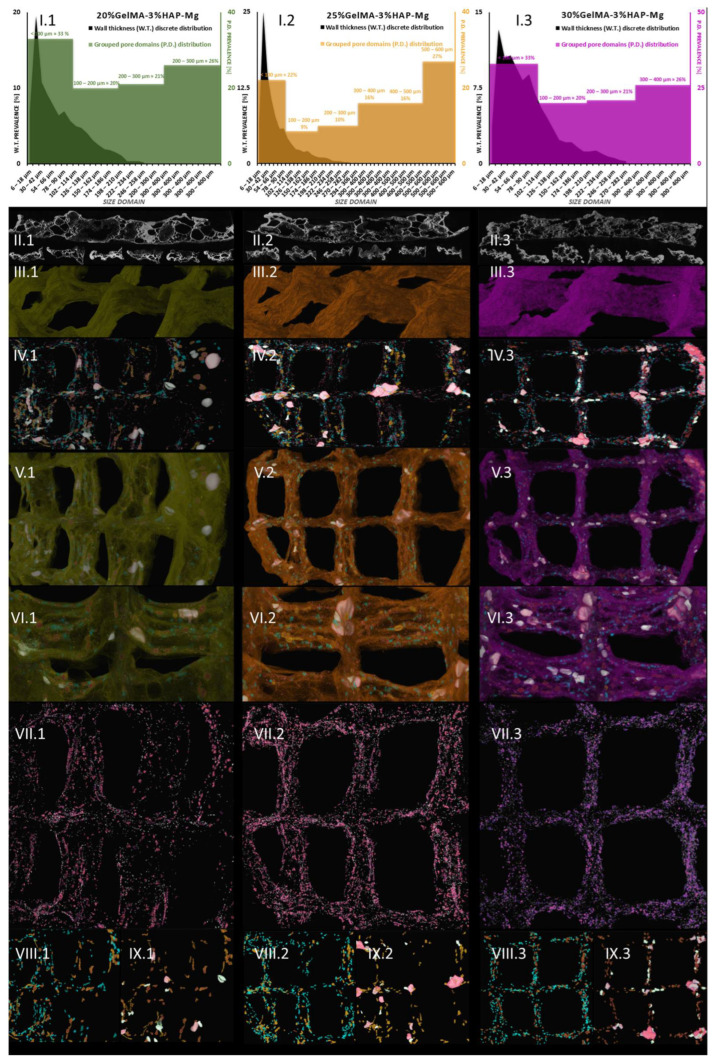
Micro-CT images of: (1 green) 20%GelMA-3%HAP-Mg, (2 orange) 25%GelMA-3%HAP-Mg, and (3 purple) 30%GelMA-3%HAP-Mg 3D printed composites.

Following this, we addressed the morphological variability of the three Zn-containing prints, aiming to consider the impact of GelMA concentration. Micro-computer tomography was used as a dual-aim mean of characterization to depict the microscopic in-volume features and the overview of the layer-by-layer fabricated objects. It was also used to measure specific traits of the specimens. Figure 14 depicts the results of the pore/wall features of the objects (I sets) and the representative cross-section in the prints (II sets), where an intricate network of pores can be observed which we specifically reconstructed as objects and superimposed with the original sample tomogram (III) to observe their distribution within the printed objects (IV and V sets). Their homogeneous distribution was favored by the very porous solid template, better displayed under higher transparency in VI subdivisions. Morphology traits of 3D-printed objects made of 20%GelMA-3%HAP-Mg, 25%GelMA-3%HAP-Mg, and 30%GelMA-3%HAP-Mg composite inks were also investigated by means of micro-computer tomography. Images and numeric data provided upon micro-CT analysis are depicted in Figure 15. For the three compositions, we scrutinized the tomograms in CTAn (subset (I)) and CTVox (subsets (II–XI)).

A quantitative analysis was performed using CTAn software to measure the solid and porous features of the prints. Figure 14(I) illustrates the charted data calculated. Discrete wall thickness incidence values were plotted against their size domains in micrometers and were superimposed with the incidence of pore domains, grouped in three spans: <100 µm, 100–200 µm, >200 µm. In Figure 14(II) subdivisions, we illustrated representative 2D sections from the tomogram, where a clear separation between the object and the pores can be observed. It is important to stress the assortment of pore sizes for all three Zn containing compositions and also the more uniform wall thickness occurring in the sample. The numerical quantification reflects accordingly these observations: in the three specimens, the walls that are formed upon freeze-drying are mostly under 100 µm, while some important share of pores span to the three-fold range. Only 4.9% of the walls in the 20%GelMA-3%HAP-Zn exceed 100 µm and a slight increase in this share was recorded for 25%GelMA-3%HAP-Zn (8.5%) and the 30%GelMA-3%HAP-Zn (13.2%), as the solid content in the printing inks increased. The largest pores within the three specimens were lodged between the ranges of 270–294 µm, but for a clearer depiction of group pore sizes, we approximated them to 300 µm. All samples have a balanced ratio between pore size and wall size since the largest pores are roughly threefold bigger than the thickest solid domains. 

Tomography images offer insight into pore orientation within the 3D printed objects. In the set of three, we can observe anisotropic pore domains outspread in all cross-sectional views, indicating that there is an important variability of extents in the pores formed during the freeze-drying process regardless of the polymer content of the ink. The same comments can be made for (II.1), (II.2), and (II.3) images, which offer insight into random mono-planar sections (long filaments/short filaments) in CTAn, where the separation between specimen walls and porosity is more discernible. The (Figure 15(II)) subsets served as a starting point for the thresholding stage of CTAn analysis, which further enabled the pores and object feature quantifications charted in Figure 15(I.1–3). 

In addition to this quantification, CTAn provided the means to reconstruct the porosity of the prints in a restricted manner. As was the case with Zn-containing composites from the original object dataset, we generated subsequent tomograms using image processing protocols that led to the reconstruction of diameter-controlled pore networks. These individual datasets can be discretely loaded and visualized for better comprehension of their spread within the prints’ walls. The (VII–XI) subsets render the reconstructed pores white/dark red → [6–100 μm], turquoise/copper → [100–200 μm], and light green/rose → [200–300 μm]. These morphological features are associated with the confining of solid filaments in four square lattices in the printed objects. In subset (IV), a distanced view of all the pores in the object is loaded, while for (V) and (VI), we overlaid the original composite print onto the pore networks for an exhaustive understanding of their interfaces. Apart from being highly porous, as it is suggestive in divisions (VI), the materials feature a proper printing fidelity to the CAM design, similar to the Mg formulations. The deposited inks kept their morpho-structural integrity during the freezing and drying stages; the scanned objects are robust, and only at the junction of the filament does more material amass naturally. According to (IV) and (IX) figures, pores larger than 300 μm appear at the crossroads of the printing head paths. However, for the rest of the pore domains we investigated, a homogeneous distribution was observed. 

This means that there is an interesting pore architecture modelling the composite matrix (Figure 14(VI) and Figure 15(VI)). By visual appraisal of the qualitative images and by precise calculation of pore/total object ration, no important differences can be highlighted between the Zn containing specimens. Total porosity (tp) barely varies with the concentration of GelMA, in this ranking: tp25%GelMA-3%HAP-Zn < tp20%GelMA-3%HAP-Zn < tp30%GelMA-3%HAP-Zn (Table 3). However, also according to Table 3, tp is higher by ∼20% when Mg is used as a doping agent instead of Zn, at equal GelMA concentrations.

After calculating the global pore features of the samples, we were able to reconstruct specific pore domains as individual objects and superimpose them in CTVox software into the objects’ tomogram and also display them separately (Figure 14(VII), distribution of the pores under 100 µm, Figure 14(VIII), pores within the range of 100–200 µm, and Figure 14(IX), 200–300 µm pores). Figure 14(III) depicts the colored tomograms of the scanned objects, constrained to four square units of the prints. They all exhibit a rough surface, indicating a large specific area for cell interaction and adhesion. Even though 25%GelMA-3%HAP-Zn and 30%GelMA-3%HAP-Zn seem to have a more rugged aspect according to the CTAn calculations (Table 3), the largest specific superficial area (including inner porosity surface) is found in 20%GelMA-3%HAP-Zn, probably due to the fact that it has the largest proportion of small sized pores with a large specific surface. Regarding the shape fidelity of these objects, traces of the deposition filaments are visible; in addition, with the exception of 20%GelMA-3%HAP-Zn which gained a curvature upon drying, the compositions with more solid content are more stable and suffer less deformation. Reconstructed pore domains (<100 µm in yellow, 100–200 µm in beige, and >200 µm in lavender) were visualized in Figure 14(IV) and added into the solid object tomograms to better understand how they are oriented therein (Figure 14(V)). In the complex illustrations of subsets V, pores seem to be evenly distributed regardless of size. Still, in order to confirm this, we portrayed the individual datasets in Figure 14(VII–IX). 

Even though osteoblasts are within the range of 10–50 μm, they develop better in scaffolds with a porosity that is at least double theirs [48]. According to some studies, 75–200 μm is ideal to encourage mineralized bone after implantation and above ∼300 µm is ideal to promote vascular constructs ingrowth [49]. In our Mg batch, these medium-sized pores have an incidence of 30–40% of the total porosity, but they are highly supported by the network of small pores which cater for the need of nutrients and metabolites exchange. Small pores (Figure 14(VII)), and those below the scanning limit of the analysis (6 µm) which cannot be depicted here, serve mostly as a conducting network for the nutrients through the volume of the scaffold. However, some studies point out the fact that in bone tissue engineering, scaffolds with open spaces of down to 70–80 µm can facilitate cell proliferations. The comparably homogeneous in-volume occurrence of pores within the range of 100–200 µm is individually rendered in VIII subsets, opposite division IX displaying pores larger than 200 µm. Pores below 20 μm create capillary forces found to improve cell attachment onto the surface and enhance penetration through pore channels where cells do not initially accommodate unless therefore stimulated [50]. Such variety of pores should be able to encompass all the requirements for proper cell colonization and functional tissue formation. At the lowest GelMa concentration, small pores are prevalent, with a share of almost 39%, while medium and large pores occupy 35 and 26% of the total pore space within (Figure 14(I.1)). The trend changes significantly for the denser compositions, where the pore share incidence exhibits a classical Gaussian distribution and the majority of internal open space is found within 100–200 µm (45% for 25%GelMA-3%HAP-Zn and 53.6% for 30%GelMA-3%HAP-Zn), clearly represented in Figure 14(I.2,I.3) charts. This domain should be more adequate for bone progenitor cells to adhere to, synthesize their specific matrix, and cluster, serving as centers for the propagation of de novo tissue. 

To sum up, 20%GelMA-3%HAP-Zn, 25%GelMA-3%HAP-Zn, and 30%GelMA-3%HAP-Zn exhibit similar morphologies and comparable pore distributions. Upon increasing the GelMA content, a slight tendency to increase the thickness of the walls was observed, but their size domain did not go beyond 162 µm and the largest have a small share of the solid content overall. Generated pores within the three scaffolds are similar both in shape as well as size, and no pores larger than 300 µm were found. Yet, the prevalence of medium-sized pores (as considered in this study) increased with the increase in GelMA content, which could favor the seeded bone progenitors in the second stage of tissue regeneration. With respect to pore and solid content appraisal in Mg-doped compositions, it is interesting to highlight the fact that their size domain distributions differ significantly (Figure 15(I.1–3)). In the three composites, most of the generated walls were found below 100 μm. The thickness distribution was similar among 20%GelMA-3%HAP-Mg, 25%GelMA-3%HAP-Mg, and 30%GelMA-3%HAP-Mg, as the profile generated is a right-skewed Gaussian bell. However, porosity has a double distribution with a maximum under 100 μm and a second one above 200/300 μm. For values below 100 μm, there is a perfect equilibrium between pores and walls as they vary in the same trend and should benefit from primal cell colony adjustment over firm substrates. For bigger domains, the variations are inversely proportionate, which benefits the propagation of angiogenesis from the larger cavities towards the less coarse pore channel lattice. Cell colonization could also be influenced first-hand by the superficial area of the scaffold, which, according to the values listed in Table 3, is larger in the case of 25%GelMA-3%HAP-Mg. Total porosity decreases with the rise in polymer content but not in a striking fashion (from ∼75% to ∼71%). Solid content also contributes to the increase in the mean wall thickness (from 72 to 85 μm), calculated by weighted average. The impact on pore magnitude, however, does not follow a linear trend. 

Figure 14 shows the µ-CT quantitative and qualitative analysis of 20%GelMA-3%HAP-Zn, 25%GelMA-3%HAP-Zn, and 30%GelMA-3%HAP-Zn printed composites. Subsections I depict the grouped pore domains and wall thickness distributions specific to the formulations above obtained by the CTAn analyses made on the 3D-printed scaffolds [37]. Subsections II cover relevant 2D cross-sectional views of the samples. Number III divisions depict 3D views of the object, sets IV show pore reconstruction in 3D, sets V show the superimposed object with reconstructed porosity, sets VI depict a transparent view of the object to enhance its high porosity, sections VII show small pore reconstruction, sections VIII show medium pore reconstruction, and sections IX depict large pore reconstruction. The colors associated with the rebuilt pore domain are yellow → [6–100 μm], beige → [100–200 μm], and lavender → [200–300 μm].

Figure 15 shows the µ-CT quantitative and qualitative analysis of 20%GelMA-3%HAP-Mg, 25%GelMA-3%HAP-Mg, and 30%GelMA-3%HAP-Mg printed composites. Subsections I depict the grouped pore domains and wall thickness distributions specific to the formulations above obtained by the CTAn analyses made on the 3D-printed scaffolds. Subsections II cover relevant 2D cross-sectional views of the samples. Number III divisions depict 3D views of the object, sets IV show pore reconstruction in 3D, sets V show the superimposed object with reconstructed porosity, sets VI depict a transparent view of the object to enhance its high porosity, sections VII show small pore reconstruction, sections VIII show medium pore reconstruction, and sections IX depict large pore reconstruction. The colors associated with the reconstructed pore domain are white/dark red → [6–100 μm], turquoise/copper → [100–200 μm], and light green/rose → [200–300 μm], and larger in the case of 25%GelMA-3%HAP-Zn. 

#### 3.3.5. Biocompatibility Evaluation of the 3D Scaffolds

- Cell viability

**Figure 16:**
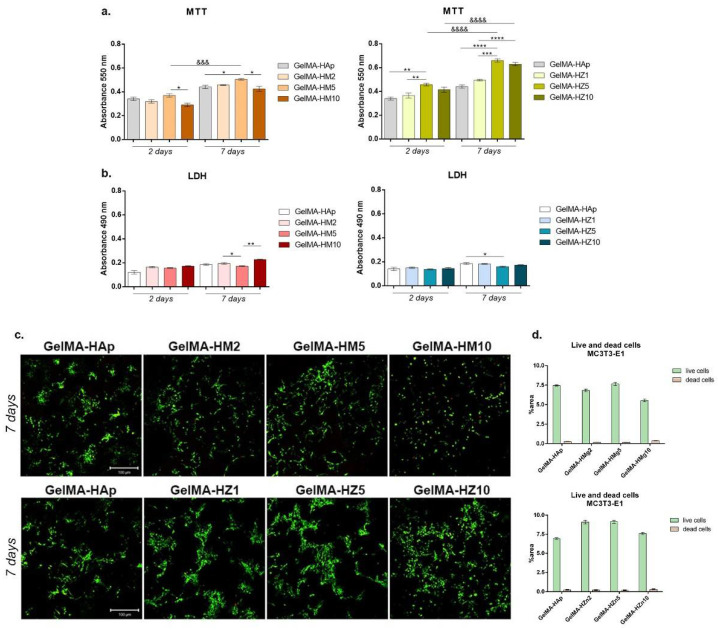
(**a**) Cell viability and proliferation profile registered after MTT test for GelMA-derived composites during one week of culture. Statistical significance: * *p* < 0.05, ** *p* < 0.01, *** *p* < 0.001, **** *p* < 0.0001, &&& *p* < 0.001, &&&& *p* < 0.0001. (**b**) Cytotoxic effect observed by employing LDH assay for the tested composites at two and seven days of culture. Statistical significance: * *p* < 0.05, ** *p* < 0.01. (**c**) Live/dead fluorescent coloration indicating with green the live cells and red the dead cells, cultured in contact with printed scaffolds (GelMA-HAp). (**d**) Quantification of green fluorescence (live cells) and red fluorescence (dead cell nuclei) levels in all composites.

A biocompatibility evaluation using an MTT test showed a good influence of cells on 3D-printed samples during one week of culture (Figure 16a). Adding magnesium ions into materials’ compositions determined the variation of cell viability in a concentration-dependent manner. After two days of seeding, a statistically significant difference between the GelMA-HM5 and GelMA-HM10 composites was noted (*p* < 0.05). This may suggest that the addition of an increased concentration of magnesium affects cell behavior and viability. After seven days, the highest rate of cell viability was supported by GelMA-HM5 in comparison with the GelMA-HAp control (*p* < 0.05). A statistically lower cell viability profile was determined in GelMA-HM10 (*p* < 0.05) compared to the effects of GelMA-HM5 on cellular responses. Additionally, cells proliferated significantly (*p* < 0.001) from two to seven days of in vitro cell culture on the GelMA-HM5 scaffold. Similarly, Bauer et al. evaluated the effects of magnesium ion content in the HAp/whitlockite scaffolds on human embryonic kidney cells, showing that all tested scaffolds were non-cytotoxic and supported cell viability and growth [43].

Scaffolds have also been enriched with different concentrations of zinc, which has improved their biocompatibility. After two days of culture, it was observed that the viability of cells was significantly higher in association with GelMA-HZ5 compared to the control sample (GelMA-HAp) and samples with zinc (GelMA-HZ1) (*p* < 0.01). 

Only slight (non-significant) differences in cell viability were noticed between GelMA-HZ5 and GelMA-HZ10. After seven days of culture, a significantly better viability was found in GelMA-HZ5 in comparison with the GelMA-HAp control (*p* < 0.0001) and GelMA-HZ1 (*p* < 0.001), which indicates a positive influence of equilibrated zinc concentrations on cell behavior. An increase in cell proliferation profiles was observed in contact with GelMA-HZ5 (*p* < 0.0001) and GelMA-HZ10 (*p* < 0.0001), supporting the hypothesis that an optimal concentration of zinc favors cell viability and proliferation. Popa et al. studied the effects of zinc-doped HA in a collagen matrix on the HeLa cell line, and concluded that the scaffold supported cell distribution and proliferation on the material’s surface [26].

- Materials’ cytotoxicity

We considered the cytotoxicity of the 3D-printed scaffold (GelMA-HAp) in association with MC3T3-E1 cells. In accordance with the MTT assay results, relatively low values of cytotoxicity were registered in all samples, indicating proper biocompatibility (Figure 16b). After two days, no statistical differences in the values of LDH were determined in magnesium enriched scaffolds, but a slight increase in the level of cytotoxicity was determined after the highest concentration of magnesium was added. After seven days, a significant increase in cytotoxicity was observed in the quantity of dead cells on printed scaffolds GelMA-HM2 and GelMA-HM10, compared to GelMA-HM5 (*p* < 0.05 and *p* < 0.01, respectively). This may suggest that a high concentration of magnesium in the GelMA-HAp scaffold composition negatively affects cell behavior and viability.

The addition of zinc in the GelMA-HAp material composition determined no statistical differences in the values of LDH after two days of culture in standard conditions. A similar profile was found after seven days, but a significant decrease in the amount of LDH released into culture media was registered in contact with GelMA-HZ5 compared with GelMA-HAp control (*p* < 0.05). These cytotoxicity profiles suggest that zinc produces the most dissipate cytotoxic effect.

- Live/dead fluorescence microscopy

Fluorescent staining was used to qualitatively measure the viability and proliferation profiles in scaffolds enriched with magnesium or zinc compared to the control scaffold (Figure 16c). The increase in magnesium concentration in the materials’ composition resulted in a lower cell viability on the surface of the materials. Additionally, a higher proportion of dead cells, marked by red fluorescence, was found in the GelMA-HM10 composite. A higher number of live cells and a tendency to group was found in contact with GelMA-HM5, in comparison with the GelMA-HAp control, thus suggesting that higher concentrations of magnesium ions can decrease cell viability and proliferation, but an adequate concentration can support cell adhesion and growth.

Noticeable results were obtained on all zinc-enriched scaffolds. Cells formed large groups on all composites, showing that these composites offer noticeable adhesion and growth conditions for cells. A moderately higher ratio of live cells and a tendency to group was noticed in GelMA-HZ5, suggesting that a 5% zinc concentration offers more favorable conditions.

Three-dimensional-printed scaffolds exhibit biocompatibility aspects, indicated by a higher proportion of living cells compared to dead ones. Although minor changes in the ability to support cell growth were noticed between the scaffolds, a higher cell density was found in samples HM5 and HZ5 compared to the GelMA-HAp control.

#### 3.3.6. Evaluation of Cellular Reaction on the Printed Scaffolds

**Figure 17:**
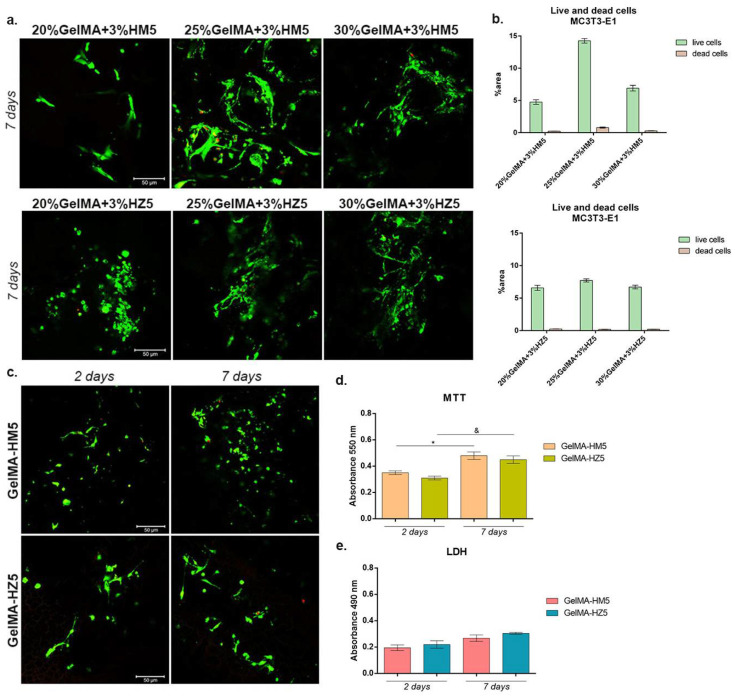
(**a**) Live/dead fluorescent coloration coupled with confocal microscopy, indicating live cells (green) and dead cell (red) put in contact with 20%, 25%, and 30% GelMA-enriched scaffolds. Scale bar size is 50 µm. (**b**) Quantification of green fluorescence (live cells) and red fluorescence (dead cell nuclei) levels in all composites. (**c**) Qualitative assessment of living cells (green) and dead cells (red) in contact with GelMA-HM5 and GelMA-HZ5 printed scaffolds. Scale bar size is 50 µm. (**d**) Biocompatibility assessment by MTT test after seven days of culture in contact with printed samples GelMA-HM5 and GelMA-HZ5. Statistical significance: */& *p* < 0.05. (**e**) Cytotoxic level registered for GelMA-HM5 and GelMA-HZ5 scaffolds indicated by LDH test at two and seven days of culture.

After cell seeding, groups of cells were noted in all magnesium-enriched compositions (Figure 17a), especially in 25% GelMA-based scaffolds, where the highest percentage of viable cells and groups were registered. It can also be noticed that due to the increase in GelMA concentration (30%), the material becomes more compact, the porosity is reduced, and due to the lower degree of porosity, the cells did not have the optimal conditions to adhere and proliferate.

In zinc-containing scaffolds, a high percentage of live cells was obtained, indicating a proper biocompatibility of all scaffolds. Moreover, the highest cell density and appreciable groups of cells were found in contact with 25% and 30% GelMA biocomposites, compared to 20% GelMA biocomposite. Even if no major differences in the capacity to support cell viability were observed between 25% and 30% GelMA biocomposites, a relatively higher cell density was indicated by 25%GelMA + 3%HZ5.

The biocompatibility evaluation of the printed scaffolds (GelMA-HM5 and GelMA-HZ5) was performed at two and seven days of culture (Figure 17c). Live/dead fluorescent staining indicated the rate among live and dead cells in the tested printed systems. It can be noticed that there was a positive rate among live and dead cells in both scaffolds. After seven days, a significant number of live cells were registered on both scaffolds, suggesting that these two composites promote cell viability and proliferation. The obtained data revealed that MC3T3-E1 cells proliferated and maintained their viability from two to seven days of culture, thus confirming MTT and LDH results. 

Much more viable cells were observed in contact with GelMA-HM5, as indicated by the results of the MTT assay compared with the GelMA-HZ5 (Figure 17d). On the other hand, after seven days, a significant profile of viable cells was found in contact with GelMA-HM5 (*p* < 0.05). The same ascending profile was registered for cells cultured for the MC3T3-E1/GelMA-HZ5 biosystem (*p* < 0.05).

The LDH assay indicated an overall low cytotoxicity of the scaffolds during seven days of culture (Figure 17e). Although slight differences between the cytotoxicity of materials could be observed during one week of culture, none of them were significant. After seven days of culture, a slight increase in cytotoxicity was observed in GelMA-HZ5, suggesting that the addition of zinc instead of magnesium could have a different cellular response, decreasing the viability and proliferation capacity.

#### 3.3.7. Osteogenic Differentiation Supported by GelMA-HM5 and GelMA-HZ5 Printed Scaffolds

**Figure 18:**
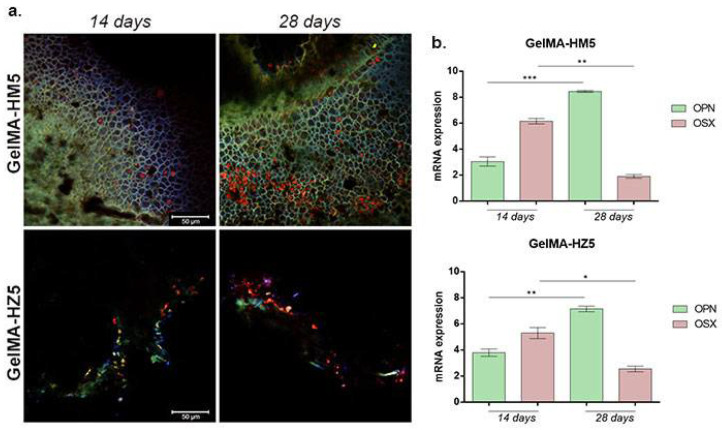
(**a**) Protein expression of osteogenic markers (OPN and OSX) after 14 and 28 days of osteogenic differentiation visualized by immunofluorescent staining in contact with GelMA-HM5 and GelMA-HZ5 composites. Murine preosteoblasts were highlighted using immunofluorescence, showing the composite of cell nuclei in blue, OPN in green, and OSX in red. Scale bar size is 50 µm. (**b**) Gene expression of OPN and OSX during 28 days of osteogenesis in the presence of GelMA-HM5 and GelMA-HZ5 scaffolds. Statistical significance: * *p* < 0.05, ** *p* < 0.01, *** *p* < 0.001.

The capacity of MC3E3-E1 to undergo osteogenesis when cultivated in contact with GelMA-HM5 and GelMA-HZ5 printed scaffolds was evaluated by immunofluorescence and qPCR. The gene and protein expression of OPN and OSX were evaluated at 14 and 28 days of osteogenic differentiation (Figure 18). 

Compared to 14 days of osteogenesis, after 28 days in contact with the GelMA-HM5 scaffold, the gene expression level of OPN had statistically significantly increased (*p* < 0.001), suggesting a positive effect on osteogenesis with the addition of magnesium in the composition of the scaffolds. The same ascending profile of OPN level was registered in contact with the GelMA-HZ5 scaffold, but at a slower rate. Moreover, OSX gene expression values were observed after 14 days, but notably decreased before 28 days in contact with both GelMA-HM5 (*p* < 0.01) and GelMA-HZ5 (*p* < 0.05). 

The results of OPN and OSX gene expression evaluations were confirmed at the protein levels, using immunofluorescence coupled with confocal microscopy. After 14 days of osteogenesis, OPN was poorly expressed on both GelMA-HM5 and GelMA-HZ5 printed scaffolds, but after 28 days of osteogenesis, the OPN expression profile increased. These findings suggest a positive effect of magnesium and zinc on the osteogenic differentiation process.

OSX fluorescent staining also confirmed the capacity of GelMA-HM5 and GelMA-HZ5 to support osteogenesis, especially in a magnesium-enriched scaffold.

## 4. Conclusions

According to the obtained results, the newly developed bio-inks based on 25%GelMA-annorganic filler and 30%GelMA-annorganic filler allowed the printing of scaffolds with defined architecture. These scaffolds could serve as a suitable medium for bone regeneration because they allow the cells to attach, spread, and proliferate. Biological studies and OPN and OSX gene expression evaluations were confirmed at the protein levels, using immunofluorescence coupled with confocal microscopy. These findings suggest a positive effect of magnesium and zinc on the osteogenic differentiation process.

OSX fluorescent staining also confirmed the capacity of GelMA-HM5 and GelMA-HZ5 to support osteogenesis, especially in a magnesium-enriched scaffold.

## Figures and Tables

**Table 1:** Values obtained by calculating the mean diameter of crystallite, D (nm) with the Debye–Scherrer equation.

Samples	Crystallite Size—D (nm)
HM2	42.59
HM5	44.13
HM10	45.71
HZ1	45.12
HZ5	41.53
HZ10	41.95

**Table 2:** The main absorption bands of GelMA and their assignments.

Wavenumbers (cm^−1^)	Assignment	Mod of Vibration	Type of Bomd
3200–3400	the amide A band	stretching vibration	N-H bond
3069	the amide B band	stretching vibration	C-H bond
1640	the amide I	-	C=O bond
1536	the amide II	stretching and deformation vibration	C-N and N-H bond
1241	the amide III	deformation vibration	N-H bond

**Table 3:** Quantitative traits of the GelMA-3%HAP-Zn and GelMA-3%HAP-Mg batches in 3D-printed objects.

Sample	Object Surface [µm^2^]	Object Surface/ Volume [µm^−1^]	Porosity [%]		Medium Wall Thickness [µm]	Medium Pore Diameter [µm]
			Total	Closed		
20%GelMA-3%HAP-Zn	3.95 × 10^9^	7.37 × 10^−2^	57.4	1.24	54	99
25%GelMA-3%HAP-Zn	2.26 × 10^9^	6.12 × 10^−2^	57.0	2.47	57	116
30%GelMA-3%HAP-Zn	2.88 × 10^9^	5.07 × 10^−2^	59.1	2.16	82	142
20%GelMA-3%HAP-Mg	3.48 × 10^9^	6.56 × 10^−2^	74.8	1.39	72	169
25%GelMA-3%HAP-Mg	5.46 × 10^9^	6.86 × 10^−2^	72.9	1.02	77	178
30%GelMA-3%HAP-Mg	4.27 × 10^9^	5.14 × 10^−2^	70.9	2.34	84	174

## Data Availability

Not applicable.
